# Metformin Treatment Is Not Associated with Altered PD-L1 Expression in Diabetic Patients with Oral Squamous Cell Carcinoma

**DOI:** 10.3390/jcm13185632

**Published:** 2024-09-23

**Authors:** Andreas Mamilos, Lina Winter, Alexander Lein, Steffen Spoerl, Nils Ludwig, Tobias Ettl, Julian Künzel, Torsten Reichert, Gerrit Spanier, Christoph Brochhausen

**Affiliations:** 1 Institute of Pathology, University of Regensburg, 93053 Regensburg, Germany; 2 Department of Pathology, German Oncology Centre, Limassol 4108, Cyprus; 3 Institute of Pathology Medical Faculty Mannheim, Heidelberg University, 68167 Mannheim, Germany; 4 Department of Otorhinolaryngology, Head and Neck Surgery, Medical University Vienna, 1090 Vienna, Austria; 5 Department of Cranio-Maxillofacial Surgery, University Hospital Regensburg, 93053 Regensburg, Germany; 6 Department of Otorhinolaryngology, University Hospital Regensburg, 93053 Regensburg, Germany

**Keywords:** oral squamous cell carcinoma, OSCC, diabetes, metformin, PD-L1, immunohistochemistry

## Abstract

**Background**: The anti-neoplastic activity of metformin is a subject of current debate. Preclinical data have suggested that metformin enhances PD-L1 anti-tumor effects in various cancer entities by decreasing insulin levels and inducing energetic stress. However, its impact on PD-L1 expression remains unclear in a clinical setting. Therefore, we aim to investigate the impact of metformin treatment in type 2 diabetes mellitus (DM) patients on PD-L1 expression in patients with oral squamous cell carcinoma (OSCC). **Methods**: We performed a retrospective analysis of patients with DM and OSCC treated at our tertiary referral center over a period of 12 years. The tumor proportion score (TPS), immune cell score (IC), and combined positive score (CPS) were used to quantify PD-L1 expression. PD-L1 expression of patients receiving metformin was compared to a control group without metformin prescription. **Results:** A total of 68 patients diagnosed with OSCC and DM were analyzed, with 24 receiving and 44 not receiving metformin therapy. No statistically significant differences were identified between the metformin and non-metformin groups for any of the scores (TPS: *p* = 0.818; IC: *p* = 0.748; CPS: *p* = 0.387). **Conclusions**: In contrast to previous studies, we could not find significant differences in PD-L1 expression between patients with and without metformin intake. Further research needs to shed light on the exact mechanism of metformin in different tumor entities. A comprehensive understanding of metformin’s role in cancer therapy could provide valuable insights for potential use of metformin as an adjuvant treatment to immune checkpoint therapy.

## 1. Introduction

Oral squamous cell carcinoma (OSCC) stands as the sixth most frequent cancer globally, constituting 90% of oral cavity tumors [1,2]. The primary etiological factors for OSCC include tobacco use, excessive alcohol consumption, and human papilloma virus (HPV) infections [3]. Furthermore, Ujpál et al. propose diabetes mellitus (DM) as a significant risk factor for oral precancerous lesions and OSCC [4]. Interestingly, patients diagnosed with DM and OSCC exhibit a reduced prognosis, characterized by lower overall survival and recurrence-free survival rates compared to non-DM patients [5].

In general, type 2 DM represents a significant global health challenge with an increasing incidence. The multifaceted management of type 2 DM involves various therapeutic modalities, with metformin assuming a crucial role as first-line medication [6]. Despite extensive research, the precise physiological mechanism of metformin, a 1,1-dimethylbiguanide within the biguanide drug class, remains incompletely understood. After phenformin and buformin were withdrawn from the market in the late 1970s due to numerous cases of severe lactic acidosis, metformin remains the only clinically relevant biguanide in the management of type 2 DM [7]. Metformin exerts its main effects on the intestines and liver, optimizing energy liberated from glycolysis in the gastrointestinal tract. This leads to a reduction in glucose absorption and an increase in hepatic lactate release [8]. The principal mechanism by which metformin reduces blood glucose levels involves its stimulatory impact on adenosine monophosphate-activated protein kinase (AMPK). This activation reflects the physiological response to metabolic stress, such as hypoxia or glucose deprivation [9].

In recent years, several studies have shown that patients taking metformin for type 2 DM have a reduced incidence and mortality of malignancy [10]. Inhibition of tumor growth has been described, for example, in patients with breast tumors or esophageal carcinoma [11,12]. The delayed identification of a direct correlation between cancer and metformin is attributed to metformin’s historical categorization as an antidiabetic agent, thereby deflecting attention away from its immediate consideration in tumor research.

In recent years, remarkable efforts have been made in the exploration of novel cancer treatments, with a specific emphasis on immune checkpoint molecules, particularly programmed death-ligand 1 (PD-L1) [13,14]. These molecules play a critical role in enabling cancer cells to evade immune detection by inhibiting T-cell activity within the tumor microenvironment. By binding to the PD-1 receptor on T-cells, PD-L1 suppresses their function, thereby promoting tumor survival and contributing to poor prognosis and resistance to conventional therapies in cancers such as melanoma, lung cancer, and OSCC [15]. Given its pivotal role in immune evasion, PD-L1 has become a major focus in cancer immunotherapy research. Currently, two PD-1 inhibitor drugs, pembrolizumab and nivolumab, are recommended as first-line therapy in the recurrent and or metastatic setting of OSCC [16]. The indication for the application of immune checkpoint therapy is determined based on histological scores that evaluate the expression of PD-1 in tumor tissue, such as the CPS score [14]. As interest grows in modulating immune checkpoints for therapeutic purposes, it is essential to explore factors influencing PD-L1 expression. One such factor is metformin, which has shown potential immunomodulatory effects, though its impact on PD-L1 expression is not yet fully understood [17,18]. For example, metformin modulates the interaction between the AMPK-mTOR and PD-L1 pathways. By activating AMPK, metformin inhibits mTOR signaling, which in turn reduces the expression of HIF-1α, a key driver of PD-L1 under hypoxic conditions. This metabolic regulation may lead to decreased PD-L1 expression, thus reducing immune evasion and enhancing the anti-tumor immune response [18,19].

The aim of this study is to investigate whether metformin is associated with decreased PD-L1 expression in OSCC. Given the clinical relevance of PD-L1 in immune checkpoint inhibition and the potential impact of metformin on the tumor microenvironment, this study aims to contribute to a better understanding of the interplay between diabetes treatment and immune modulation in OSCC.

## 2. Materials and Methods

### 2.1. Study Design and Patient Cohort

This retrospective cohort study included adult patients diagnosed with diabetes mellitus and a primary diagnosis of OSCC. The study was conducted at the Departments of Cranio-Maxillofacial Surgery and Otorhinolaryngology at University Hospital Regensburg, Germany. Between January 2005 and December 2017, a total of 90 patients were identified from electronical hospital records. Of these, a total of 68 patients met the inclusion criteria and were included in the final analysis. The inclusion criteria were (1) adult patients aged ≥ 18 years; (2) diagnosed with both diabetes and primary OSCC; (3) underwent curative resection without prior neoadjuvant treatment; (4) negative margins on tumor resection; (5) concurrent cervical lymph node dissection confirmed by clinical and radiologic examination; and (6) had high-quality histological slides available for analysis. Exclusion criteria included a history of cervical lymph node dissection or prior radiotherapy/radio-chemotherapy for OSCC. Patient characteristics, including age, gender, smoking history, and alcohol consumption, as well as clinical data, were collected. Tumor staging was performed according to the “TNM classification of malignant tumours”, published by the Union for International Cancer Control (UICC) in its 7th edition [20]. Metformin use was withdrawn from archived digital and paper-based patient records. However, due to the retrospective acquisition of data, the detailed timespan and dosage of preoperative metformin were not available.

### 2.2. Tissue Samples and Immunohistochemistry

**Figure 1:**
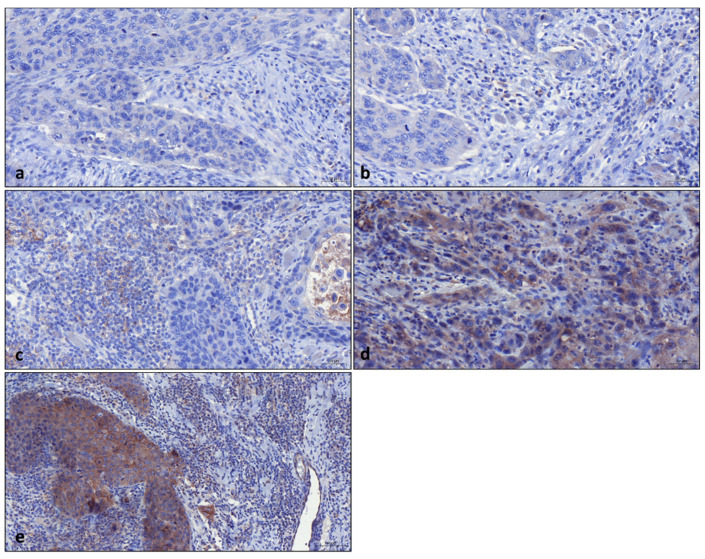
Representative images for combined positive score (CPS) evaluation: (**a**) Group with CPS 0, (**b**) group with CPS >0–<1, (**c**) group with CPS ≥1–<20, (**d**) group with CPS ≥20–<50, (**e**) group with CPS ≥50–100. All samples are shown at 200× magnification.

Formalin-fixed, paraffin-embedded tissues were retrieved from the archive of the Institute of Pathology, University of Regensburg, Germany. Two experienced head and neck pathologists (A.M., C.B.) reviewed all newly prepared hematoxylin and eosin (HE) sections from all candidate specimens to select the optimal slides. A total of 68 diabetic OSCC patients could be enrolled in the study. All immunohistochemical stains were performed on tissue sections prepared from formalin-fixed paraffin-embedded tissue blocks. Tumor tissues were sectioned into 2–3 µm slices. All tissues were fixed in 4% neutral-buffered formalin. The procedure was part of the established routine diagnostics, as described previously [21,22]. For in situ characterization, standard routine diagnostic procedures and antibodies were applied. Then, immunohistochemical reactions for PD-L1 (Dako Anti-Human PD-L1 Clone 22C3) were performed. Briefly, immunohistochemical staining was performed using a Roche Ventana Benchmark Ultra automated slide stainer (Ventana Medical Systems, Roche, France) with the OptiView DAB IHC Detection Kit (Roche, France). All slides were scanned (3DHISTECH Ltd. Pannoramic slide scanner 250, Budapest, Hungary) and evaluated using virtual microscopy software (3DHISTECH Ltd. Case Viewer Ver.2.2, Budapest, Hungary). For PD-L1, three common diagnostic scores (tumor proportion score (TPS), immune cell score (IC), and combined positive score (CPS)) were assessed according to the guidelines by two experienced pathologists as previously described [23,24]. Representative images for the assessment of the CPS score are presented in Figure 1.

### 2.3. Statistical Methods

Normal distribution of continuous variables was assessed by employing the Shapiro–Wilk test. Parametric values underwent the *t*-test, while nonparametric values were analyzed by the Mann–Whitney U test. In clinical practice, PD-L1 scores such as CPS or TPS are routinely used as categorical variables to guide treatment decisions. Subsequently, the diagnostic scores were grouped as follows: <1, ≥1–<20, ≥20–<50, ≥50–100. The subdivision was performed based on the designated cut-off values that indicate the appropriateness of immune checkpoint therapy. Notably, scores of 0, 1, 20, and 50 hold significance. Categorial variables were examined through Pearson’s chi-squared test. In cases where one or more cell counts fell below five, Fisher’s exact test was used. Any *p*-values ≤ 0.05 were considered statistically significant. All analyses and visualizations were performed using STATA (StataCorp. 2023. Stata Statistical Software: Release 18. College Station, TX, USA: StataCorp LLC.) and GraphPad Prism (version 10.0.0 for Mac OS X, GraphPad Software, Boston, MA, USA).

### 2.4. Ethics

The protocol was approved by the University of Regensburg Ethics Committee (Number: 12-101-0070) and it was conducted in accordance with the ethical standards of the declaration of Helsinki.

## 3. Results

A total of 68 patients with diagnosed OSCC and DM type 2 were examined and divided into two groups based on their treatment regimen: those who received metformin (*n* = 24; 35.29%) and those who did not (*n* = 44; *p* = 64.71%). The mean age was 63.6 years, with 68.2% and 66.7% of the patients being younger than 70 years in the non-metformin and metformin groups, respectively (*p* = 0.898). Furthermore, gender distribution was similar between groups, with males comprising 72.7% of the non-metformin group and 79.2% of the metformin group.

Additionally, the analysis of risk factors such as smoking, and alcohol consumption revealed no significant differences between the two groups. A total of 72.7% of the non-metformin group and 54.2% of the metformin group were smokers (*p* = 0.122). Correspondingly, alcohol abuse was observed in both groups (68.2% in non-metformin vs. 66.7% in the metformin group; *p* = 0.898).

In the non-metformin group, the most common primary site was tongue (*n* = 14; 31.8%), whereas the floor of the mouth (*n* = 8; 33.3%) was the most frequent primary site in the metformin group. Overall, no significant differences regarding primary site were found (*p* = 0.528). The TNM classification showed no significant differences in tumor size (T) and nodal stage (N) between the groups (T: *p* = 0.798, N: *p* = 0.658). The significant difference in terms of metastatic spread (*p* < 0.001) results from a higher proportion of patients with unknown metastatic status in the metformin group (37.5%, compared to none in the non-metformin group). Also, there were no significant differences regarding perineural (*p* = 0.819), lymph vessel invasion (*p* = 0.857), and blood vessel invasion (*p* = 0.289). Detailed clinicopathological characteristics are listed in Table 1.

Overall, the PD-L1 expression across all observed clinicopathological scores (IC, TPS, CPS) did not differ significantly between those treated with or without metformin (IC: *p* = 0.748; TPS: *p* = 0.818; CPS: *p* = 0.387).

### 3.1. Immune Cell Score (IC)

In the non-metformin group, 7 patients (15.9%) had a score of <1, 27 (61.4%) had scores between ≥1–<20, 9 (20.5%) had scores between ≥20–<50, and 1 (2.3%) had a score between ≥50–100. In the metformin group, 3 patients (12.5%) had a score of <1, 17 (70.8%) had scores between ≥1–<20, 3 (12.5%) had scores between ≥20–<50, and 1 (4.2%) had a score between ≥50–100. The *p*-value was 0.748, indicating no significant difference between the groups.

### 3.2. Tumor Proportion Score (TPS)

For the non-metformin group, 23 patients (52.3%) had a score of <1, 11 (25.0%) had scores between ≥1–<20, 5 (11.4%) had scores between ≥20–<50, and 5 (11.4%) had scores between ≥50–100. For the metformin group, 15 patients (62.5%) had a score of <1, 6 (25.0%) had scores between ≥1–<20, 1 (4.2%) had a score between ≥20–<50, and 2 (8.3%) had scores between ≥50–100. The *p*-value was 0.818, indicating no significant difference between the groups.

### 3.3. Combined Positive Score (CPS)

In the non-metformin group, 5 patients (11.4%) had a score of <1, 21 (47.7%) had scores between ≥1–<20, 11 (25.0%) had scores between ≥20–<50, and 7 (15.9%) had scores between ≥50–100. In the metformin group, 3 patients (12.5%) had a score of <1, 15 (62.5%) had scores between ≥1–<20, 2 (8.3%) had scores between ≥20–<50, and 4 (16.7%) had scores between ≥50–100. The *p*-value was 0.387, indicating no significant difference between the groups.

**Figure 2:**
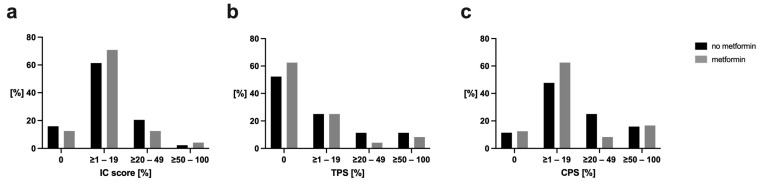
Comparison of PD-L1 scores between the metformin and non-metformin study groups. (**a**) IC, (**b**) TPS, and (**c**) CPS are shown. Abbreviations: IC, immune cell score; TPS, tumor proportion score; CPS, combined positive score.

The detailed distribution of the specific scores and their designated cut-off values are displayed in Table 2 and Figure 2.

## 4. Discussion

This study compared the TP, IC, and CP scores of PD-L1 between patients taking metformin and those not taking metformin, revealing no significant differences in PD-L1 receptor expression between the two groups. These findings suggest that metformin might not influence PD-L1 expression in OSCC patients with diabetes. Since the PD-L1 score is used to select patients for therapy with PD-1 monoclonal antibodies such as pembrolizumab and nivolumab, the study demonstrated that metformin might not act as a confounder in this context.

In the complex interplay of tumorigenesis and progression, the immune system plays a crucial role. The current literature emphasizes diverse roles of metformin in different cancer types. In a recent study, Wabitsch et al. clearly demonstrated that metformin intake can increase T-cell metabolism, ultimately resulting in an improved immune response in mice with hepatocellular carcinoma (HCC) [25].

In contrast, the study by Munoz et al. showed that metformin had no influence on PD-1 expression of tumor-infiltrating CD8+ lymphocytes in a mouse model of OSCC [26]. Moreover, metformin had no effect on PD-L1 expression of the OSCC tumors, indicating a minor impact of metformin on the PD-1/PD-L1 axis [26]. These findings are in line with our observations of PD-L1 expression presented in this study. Although no difference in PD-L1 expression was observed, the question remains whether these diabetic patients respond differently to immune checkpoint blockade (ICB). In the recurrent and metastatic setting, it is known that some patients benefit from ICB even if they are PD-L1 negative [16]. For example, in the KEYNOTE-012 study, patients with PD-L1 expression < 1% treated with pembrolizumab had a response rate of 4%, compared to 22% for patients with PD-L1 expression ≥ 1% [27]. Furthermore, neoadjuvant ICB in locoregionally advanced HNSCC led to major pathological response in both PD-L1-positive and -negative patients [28]. Future studies should investigate therapy response in diabetic patients taking metformin or not.

In addition, the identification of the respective signaling pathway in each tumor entity is of high relevance. In a model of triple-negative breast cancer, Wang et al. reported that metformin influences PD-L1 expression through the JNK signaling pathway, that has regulatory effects on the immune microenvironment [17]. Moreover, Cha et al. investigated the link between PD-L1 and metformin in breast cancer in animal models. They demonstrated reduced PD-L1 expression upon metformin administration [18]. The authors found that metformin enhances the activity of immune cells, specifically cytotoxic T lymphocytes. This effect is attributed to AMP kinase activation by metformin, which then leads to phosphorylation of PD-L1, altering its structure. Consequently, PD-L1 is impeded from exiting the Golgi apparatus of tumor cells, resulting in reduced expression on the cell membrane. The diminished PD-L1 concentration on the cell surface is linked to decreased inhibition of defense cells, leading to increased apoptosis of tumor cells by the immune system [18]. However, this effect was only observed in immune-competent mice. It remains unclear whether this effect is present or functional in OSCC. The study conducted precise animal experiments with defined dosages and administration intervals, essential factors, according to Turnheimer et al., for assessing metformin’s impact [29]. However, in the present study, these parameters are not consistent, as patient dosage and administration intervals are unknown, indicating the need for future studies investigating whether the dosage or administration interval affects PD-L1 expression in OSCC.

Interestingly, our findings did not show a significant difference in PD-L1 expression, which diverges from several studies suggesting that metformin can enhance the effectiveness of PD-L1 targeted therapies in cancer treatment. For instance, Zhao et al. demonstrated that metformin enhances the efficacy of anti-PD-L1 therapy in a lung cancer mouse model through the modulation of gut microbiota [30]. Investigating the gut microbiota as a mediator of metformin’s effects on systemic and tumor immunity in diabetic OSCC patients could clarify whether a similar mechanism exists or if metformin’s impact on PD-L1 expression in OSCC is more complex.

Clinical data underline these experimental *in-vivo* results. Henderson et al. investigated the effects of metformin on patients with colorectal and lung cancers. Specifically, colorectal cancer patients on metformin had fewer deaths, recurrences, and metastases, and both colorectal and lung cancer patients showed improved 5-year and overall survival rates [31]. The authors speculated that differences in metabolic pathways between colorectal and lung cancers could account for the variations in the effects of metformin. For instance, mutations in the *LKB1* gene, upstream of AMPK and commonly mutated in lung cancer, could affect metformin’s effectiveness [31]. On the other hand, the preclinical data of Wabitsch et al. could not be confirmed in a clinical validation study in which metformin use was not associated with enhanced survival in patients with HCC receiving immunotherapy [25,32].

Our study has several limitations. First, the retrospective design of the study conducted at a single institution introduces certain constraints. This study’s retrospective design limits the ability to establish causal relationships between metformin use and PD-L1 expression. Also, the lack of longitudinal data restricts our ability to assess long-term effects or progression over time. The inclusion of patients with unknown spread status may introduce some uncertainty in interpretation, though this variable is not expected to directly influence PD-L1 expression. Additionally, the relatively small sample size of 68 patients may limit the generalizability of our findings. Future prospective, multicenter studies with larger cohorts are needed to confirm these findings and to further elucidate the underlying mechanisms more comprehensively. However, this study adds a valuable aspect to the field, questioning the positive impact of metformin stated by other researchers [33,34]. By utilizing methods used for real-world clinical decision making regarding treatment choice, our study has direct clinical relevance. Lastly, due to the retrospective data acquisition, detailed timespan and dosage data for preoperative metformin use was not available. In this context, drug concentration at the site of action plays a critical role, as a minimal concentration must be present in the surrounding tissue to achieve a corresponding effect. In this context, there exists a direct proportionality between the drug concentration at the site of action and the resulting pharmaceutical effect [29]. However, given the standard dosages prescribed by medical consultants and the regular medical care received by most diabetic cancer patients, it is assumed that adequate metformin concentrations are prevalent in our cohort.

## 5. Conclusions

Our study did not reveal a significant difference in PD-L1 receptor expression among diabetic OSCC patients receiving metformin therapy compared to those not receiving it. Interestingly, our results diverge from research in other cancer entities, such as colon and esophageal cancer, where significant differences in PD-L1 expression were observed [35,36]. This raises the question regarding the mechanism of action responsible for reduced PD-L1 expression in colon cancer, which may either not exist or be non-functional in OSCC. Further studies and experimental approaches including downstream assays are warranted to identify the exact molecular mechanism of metformin in cancer patients. A comprehensive understanding of metformin’s role in cancer therapy could offer valuable insights for future precision medicine treatment strategies, benefiting both diabetic and non-diabetic cancer patients.

## Figures and Tables

**Table 1:** Clinicopathological characteristics of OSCC patients according to metformin intake (*n* = 68) (UICC 7th edition).

	Total	No Metformin		Metformin		
Variable/Category	n	n	(% total)	n	(% total)	*p*-value
Number of patients	68	44	(100.0%)	24	(100.0%)	
Age						
≥70	22	14	(31.8%)	8	(33.3%)	
<70	46	30	(68.2%)	16	(66.7%)	*p* = 0.898
Gender						
male	51	32	(72.7%)	19	(79.2%)	
female	17	12	(27.3%)	5	(20.8%)	*p* = 0.558
Smoking history						
never	23	12	(27.3%)	11	(45.8%)	
smoker	45	32	(72.7%)	13	(54.2%)	*p* = 0.122
Alcohol abuse						
no	22	14	(31.8%)	8	(33.3%)	
yes	46	30	(68.2%)	16	(66.7%)	*p* = 0.898
Primary site						
buccal mucosa	9	6	(13.6%)	3	(12.5%)	
upper alveolus	2	0	(0.0%)	2	(8.3%)	
lower alveolus	11	8	(18.2%)	3	(12.5%)	
hard palate	7	5	(11.4%)	2	(8.3%)	
tongue	20	14	(31.8%)	6	(25.0%)	
floor of the mouth	19	11	(25.0%)	8	(33.3%)	*p* = 0.528
T						
1	23	16	(36.4%)	7	(29.2%)	
2	23	13	(29.5%)	10	(41.7%)	
3	9	6	(13.6%)	3	(12.5%)	
4	13	9	(20.5%)	4	(16.7%)	*p* = 0.798
N						
0	11	9	(20.5%)	2	(8.3%)	
1	0	0	(0.0%)	0	(0.0%)	
2	11	7	(15.9%)	4	(16.7%)	
3	6	4	(9.1%)	2	(8.3%)	*p* = 0.658
M						
unknown	9	0	(0.0%)	9	(37.5%)	
no spread	50	37	(84.1%)	13	(54.2%)	
any spread	9	7	(15.9%)	2	(8.3%)	*p* < 0.001
Grade						
1	2	1	(2.3%)	1	(4.2%)	
2	58	36	(81.8%)	22	(91.7%)	
3	8	7	(15.9%)	1	(4.2%)	*p* = 0.335
Stage						
I	17	11	(25.0%)	6	(25.0%)	
II	12	7	(15.9%)	5	(20.8%)	
III	13	9	(20.5%)	4	(16.7%)	
IV	26	17	(38.6%)	9	(37.5%)	*p* = 0.968
Perineural invasion						
no	63	41	(93.2%)	22	(91.7%)	
yes	5	3	(6.8%)	2	(8.3%)	*p* = 0.819
Lymph vessel invasion						
no	53	34	(77.3%)	19	(79.2%)	
yes	15	10	(22.7%)	5	(20.8%)	*p* = 0.857
Blood vessel invasion						
no	66	42	(95.5%)	24	(100.0%)	
yes	2	2	(4.5%)	0	(0.0%)	*p* = 0.289

**Table 2:** Distribution of PD-L1 group-scores within OSCC patient groups with or without metformin use ^a^.

		No Metformin		Metformin		
PD-L1 score		n	(% total)	n	(% total)	*p*-value
IC						
	<1	7	(15.9%)	3	(12.5%)	
	≥1–<20	27	(61.4%)	17	(70.8%)	
	≥20–<50	9	(20.5%)	3	(12.5%)	
	≥50–100	1	(2.3%)	1	(4.2%)	*p* = 0.748
TPS						
	<1	23	(52.3%)	15	(62.5%)	
	≥1–<20	11	(25.0%)	6	(25.0%)	
	≥20–<50	5	(11.4%)	1	(4.2%)	
	≥50–100	5	(11.4%)	2	(8.3%)	*p* = 0.818
CPS						
	<1	5	(11.4%)	3	(12.5%)	
	≥1–<20	21	(47.7%)	15	(62.5%)	
	≥20–<50	11	(25.0%)	2	(8.3%)	
	≥50–100	7	(15.9%)	4	(16.7%)	*p* = 0.387

^a^ Abbreviations: IC, immune cell score; TPS, tumor proportion score; CPS, combined positive score.

## Data Availability

Data are contained within the article.
